# Cable Cars to the Nucleus: TM4SF1-Enriched Microdomains Conduct Signaling in Endothelial Cells for Blood Vessel Formation

**DOI:** 10.3390/ijms262110491

**Published:** 2025-10-29

**Authors:** Shou-Ching Jaminet

**Affiliations:** Angiex Inc., 150 Cambridge Park Drive, Cambridge, MA 02140, USA; sjaminet@angiex.com

**Keywords:** TM4SF1, TMEDS, nanopodia, endothelial cell signaling, VEGF-A, angiogenesis, blood vessel development, tetraspanin

## Abstract

Endothelial cell proliferation, migration, and intercellular interactions for blood vessel formation require coordinated signaling by a myriad of molecules. Following endothelial cell activation by growth factors and cytokines, a variety of signaling molecules are activated on the surface and transported intracellularly by TM4SF1-enriched microdomains (TMEDs), 100–300 nm diameter protein–lipid complexes recruited by the transmembrane protein TM4SF1. TMEDs internalize via microtubules from the cell surface toward the microtubule-organizing center (MTOC) and then enter the nucleus via nuclear pores (see Graphic Illustration). This internalization pathway permits delivery of activated proteins and other signaling molecules from the cell surface to the nucleus, which directly translates extracellular stimuli to modulation of gene expression. Molecules transported by this route include phospholipase C, gamma 1 (PLCγ1), histone deacetylase 6 (HDAC6), and importins. In the absence of TMEDs, endothelial cells lose the ability to divide into cultures in vitro and to support blood vessel formation in mouse embryos in vivo. We liken TMEDs to cable cars, which take in passengers at the cell surface, travel along microtubule cables, and deliver their passengers to various locations, including the “city center”, the nucleus. This commentary aims to elucidate the functions of TMEDs in endothelial cells, to show that cells, like busy cities, need efficient transport systems to deliver molecules to the destinations where they perform their cellular functions. TMEDs offer a novel and curated transport system providing selected molecules with access to the nucleus.

## 1. TM4SF1 and Its Expression—A Concise Summary

Transmembrane-4 L Six Family member 1 (TM4SF1) is a small integral membrane glycoprotein structurally related to genuine tetraspanins [1,2,3]. TM4SF1 was the first to be discovered out of thirty-nine proteins with tetraspanin topology. TM4SF1 is now sub-grouped with five other proteins into the atypical tetraspanin of the L6 family as they lack CCG-domains in extracellular loop-2 [3].

TM4SF1 was reported as a tumor-associated antigen recognized by the antibody L6 in 1986 [4]. TM4SF1 expression is generally high in cancer cells of epithelial origin [4,5], including cancer stem cells [5,6], but it is not expressed in cancer cells of hematopoietic origin according to The Cancer Cell Line Encyclopedia [7] as well as our unpublished data.

TM4SF1’s biology was largely unknown until our identification of the protein as an endothelial cell biomarker, reported in 2009 [8]. We demonstrated that TM4SF1 expression is universally high when endothelial cells are activated for proliferation and migration [8,9]. Apart from bone marrow-derived mesenchymal stem cells, TM4SF1 is either weakly (e.g., fibroblasts and smooth muscle cells) or not expressed (epithelial cells and myocytes) in other primary cell types in vitro [1,10,11]. Leukocytes and lymphocytes also do not express TM4SF1 [12]. TM4SF1 immunohistochemical staining in normal tissues in vivo is consistent with the expression patterns observed in cultures in vitro, with blood vessels being the primary location of positive signals [8,12].

### TM4SF1-Enriched Microdomain (TMED) Formation and Cellular Localization—An Introduction of TM4SF1’s Functional Activities

**Figure 1:**
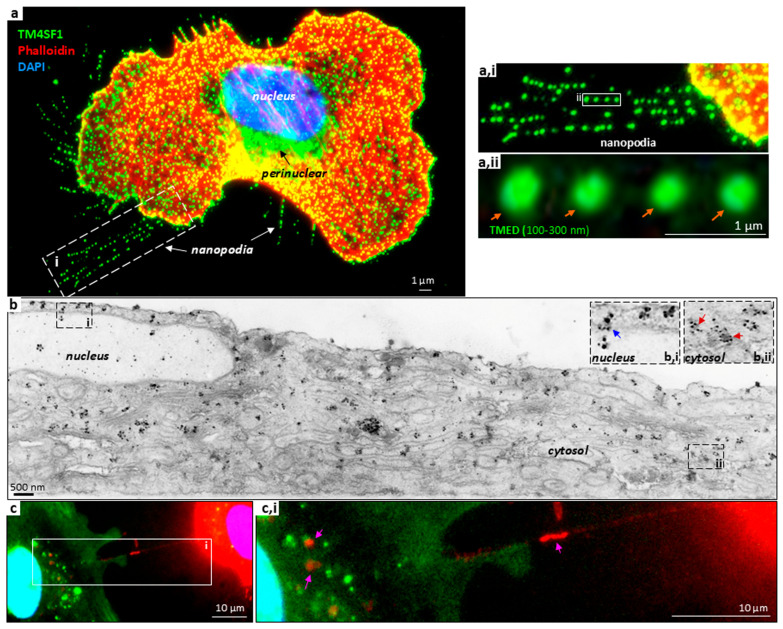
TMED formation and cellular localization in endothelial cells. (**a**) TMEDs on the endothelial cell surface are marked by green-labeled TM4SF1-directed antibodies [8]. On the luminal side, cell sur-face TMEDs internalize along microtubules to the perinuclear region [(**a**), black arrow points to perinuclear TM4SF1] [1,13]. (**b**) Electron microscopy using nanogold-labeled TM4SF1-directed an-tibodies shows TMED entering the nucleus via nuclear pores [inset (**b,i**) blue arrow indicates a point of nuclear entry; inset (**b,ii**) red arrows indicate cytosolic TMEDs] [13]. On the abluminal side, TMEDs help endothelial cells anchor to the matrix, and form thin membrane channels called na-nopodia [white arrows in (**a**) and orange arrows in (**a,ii**) that are characterized by regularly spaced TMEDs] [10]. These nanopodia extend from the leading front or trailing rear and can host actin filaments [10]. (**c**) Nanopodia develop into nanotubes to connect neighboring cells and enable in-tercellular trafficking of cytosolic components [(**c,i**), pink arrows indicate mitochondria in red be-ing transferred from MitoTracker-labeled endothelial cells to GFP-labeled endothelial cells. Some mitochondria have already arrived at the recipient cell and others are traversing a nanotube] [10,11].

Like genuine tetaspanins, TM4SF1 clusters in 100–300 nm diameter microdomains (TM4SF1-enriched microdomains or TMEDs) when its expression is high (Figure 1a,ii) [8]. However, unlike the microdomains formed by genuine tetraspanins, TMEDs traffic from the cell surface to the nucleus via microtubules, as depicted in the Graphic Illustration and the experimental data in Figure 1 [1,13]. This trafficking pathway is essential for blood vessel formation [12].

On the luminal side of endothelial cells, TMEDs serve a transport function. Recruitment of proteins to TMEDs on the cell surface (Figure 1a; orange arrows in Figure 1a,ii) triggers interactions with tubulin and internalization along microtubules [13]. Upon approaching the microtubule organizing center (MTOC; site of microtubule nucleation and assembly) (Figure 1a, black arrow), TMEDs subsequently enter the nucleus (Figure 1b; blue arrow in Figure 1b,i) [13]. TMEDs (tracked through TM4SF1-directed antibodies via fluorescent and electron microscopy) can be seen in various cellular compartments. Two hours after the antibody internalization, some TMEDs have already entered the nucleus while others are transiting the nuclear pore (Figure 1b,i, blue arrow) or traversing the cytosol (Figure 1b,ii, red arrows) [13]. If TM4SF1’s final destination is the nucleus, then the time required for a TMED to reach the destination may be determined by its original location on the plasma membrane [13]. TMED that originates above the nucleus or MTOC would travel a short distance, while TMED initiated on the cell periphery would travel a longer distance. As endothelial cells in cultures in vitro are immobile, the ability of TMEDs to reach the nuclear compartment may also be affected by the state of cellular polarization [10].

On the abluminal side of endothelial cells, TMEDs are associated with integrin-α5β1 and are readily observed in nanopodia (Figure 1a, white arrows) [8]. Nanopodia are the thin membrane channels on the leading front and the trailing rear of mobile endothelial cells that can serve as membrane hosts for actin filament activities, including filopodia extension and retraction fiber retrieval [8,10,11]. They were so named because nano represents the width which is determined by the size of TMEDs at 100 to 300 nm, whereas podia indicate a foot-like protrusion from the plasma membrane, respectively. In accordance, the reported diameters for filopodia and retraction fibers are 100–300 nm [14,15]. Once nanopodia are anchored to the matrix, the thin membrane channels do not retreat into the cells, and are left behind as cells move, in the form of a litter of membrane debris that contains cytosolic components [10,16]. Cellular structures similar to nanopodia, but organized by genuine tetraspanins, have been described as migrasomes [17,18,19].

Nanopodia participate in the regulation of endothelial cell movement by sensing cellular surroundings, detecting neighboring cells, and engaging in intercellular interaction, communication, and junction formation [10,16]. Homotypic endothelial cell interactions facilitate the formation of vascular tubes for junction formation, while heterotypic endothelial–mesenchymal stem cell interactions stabilize the forming blood vessels [10,16]. Endothelial–tumor cell interactions assist tumor cell extravasation and intravasation for metastasis (unpublished data). Nanopodia can form intercellular connections to become nanotubes, which allow the exchange of cytosolic materials between cells, including mitochondria (Figure 1c,i, pink arrows) [10].

Endothelial cells lose the ability to polarize when TM4SF1 is knocked down in cultures in vitro, rendering the cells unable to execute cytokinesis or form junctions. The greater the degree of TM4SF1 knockdown, the more severe the effect [8]. Harvesting TM4SF1 knockdown cells via trypsin is challenging, as many will be left behind. Returning TM4SF1 knockdown cells back to culture is also difficult, as they cease to adhere easily. TM4SF1 overexpression leads endothelial cells to an uncontrolled projection of nanopodia in all directions, causing them to detach from the culturing matrix; the higher the TM4SF1 expression, the easier the detachment [10,16].

Global homozygous TM4SF1 knockout in mice in vivo leads to an avascular phenotype that is lethal by embryonic day nine [12]. Heterozygous TM4SF1 embryos are smaller in body size during the early embryonic developmental stage, and about fifty percent experience a lethal brain hemorrhage by embryonic day 17 [12]. Surviving heterozygous embryos are born alive with normal physiology, including fertility, though those mice cannot execute proper wound healing and exhibit delayed tumor growth [1].

## 2. TMED-Mediated Cell Signaling

How does TM4SF1 execute its profound activities in the regulation of endothelial cell function during normal and pathological blood vessel development?

Tetraspanins share a common overall architecture of four transmembrane domains separated by two extracellular loops and one intracellular loop [20,21]. The cone-shaped transmembrane domain contains an intramembrane binding pocket that is known to recruit lipids and proteins, and enables the formation of microdomains with the assistance of extracellular loop-2 [22]. Through recruited proteins, such as integrins, enzymes, members of the immunoglobulin superfamily, receptors, and associated signaling molecules, genuine tetraspanins critically participate in signal transduction to regulate a variety of cellular functions, including cell migration, protein trafficking, and membrane integrity maintenance [20,21,22,23].

TM4SF1 has low protein homology with genuine tetraspanins. Even within its own L6 family, the highest protein homology with TM4SF1 is TM4SF4 at 50.8% (search engine: NCBI protein blast). Hence, the protein topology shared by TM4SF1 and the genuine tetraspanins is likely to be the common feature that enables recruitment of proteins and lipids to form microdomains.

Owing to post-translational modifications (glycosylation, phosphorylation, etc.), the molecular weight of the mature TM4SF1 protein of 202 amino acids is 28 kD [1,10] with a predicted globular diameter of 2–4 nm [24]. If TMEDs contain three to fourteen TM4SF1 molecules [10], then TMEDs are predominantly occupied by recruited molecules.

What proteins are recruited to TMEDs? Three observations in endothelial cells provide clues [1,8,12]: (I) TMEDs internalize via microtubules from the cell surface to the nucleus; (II) when TMEDs are depleted through TM4SF1 knockdown in vitro, microtubules become hyperacetylated and unable to respond to vascular endothelial growth factor-A (VEGF-A) for cell proliferation or migration; (III) TM4SF1 knockout mouse embryos are avascular. Given these observations, signaling molecules recruited to TMEDs are likely (i) to interact with microtubules and regulate acetylation (αTubulin and HDAC6), (ii) to support formation of filopodia (IQGAP1, β-actin, and myosin-10), (iii) to support endocytosis (dynamin and clathrin), (iv) to facilitate nuclear entry (importin-β), and (v) to transmit signals downstream from VEGFR2 for endothelial cell proliferation (PI4K, PLCγ1, PKCα, MEK1/2, and ERK) and migration (PTEN, PI3Kα,β,p85, Rac1, Akt, and PDK1) [25,26]. Fourteen (those underlined) out of the eighteen signaling molecules listed above are found in association with TMEDs on the endothelial cell surface [1,10,13]. Twelve of these fourteen—the exceptions being PKCα and PI4K—remain in association with TMEDs two hours after internalization [1]. Among membrane proteins, integrin-α5β1 is associated with TMEDs in nanopodia [8], while the genuine tetraspanin CD9 selectively appears in some TMEDs [10]. In tumor cells, others have demonstrated that TM4SF1 interacts with membrane proteins like genuine tetraspanins (CD81, CD151, CD63 [27], and CD13 [28]) and signaling proteins like DVL2 [29], SITAC [30], and DDR1 [31].

Blood vessel development is known to essentially depend on VEGFR2 activation of phospholipase C, gamma 1 (PLCγ1) [32,33,34]. Mice that lack functional PLCγ1, either through global PLCγ1 [33] or VEGFR2 knockout [35], or mutating the PLCγ1 activation domain in VEGFR2 [34], are avascular and suffer early embryonic lethality [32]. If PLCγ1 signaling is contingent on TMED-mediated transport in endothelial cells, then the avascular phenotype of TM4SF1 knockout mice is not surprising [12]. VEGFR2 is not recruited to TMEDs [1]; thus it is likely that PLCγ1 is shuttled to TMEDs after its activation by VEGFR2. The observation that TM4SF1-heterozygous embryos have a smaller body size during early embryonic development, and that almost half die in utero through intracranial hemorrhage before embryonic day 17, suggests that the inefficient transduction of signaling by an insufficient formation of TMEDs may be the underlying reason [12].

### TMEDs as Cable Cars Trafficking from the Cell Surface to the Nucleus

We compare TMEDs to cable cars and signaling molecules to their passengers (see the Graphic Illustration). TMEDs are assembled on the cell surface (city wall). Recruitment of certain proteins may lead TMEDs to stay on the city wall for the formation and extension of nanopodia (expeditionary parties), while recruitment of others will trigger intracellular transport of TMEDs by connecting TMEDs to microtubules (cables) and facilitating their entry into the intracellular space (city). Traveling along the cables allows TMEDs to circumvent intracellular membrane components (buildings), arrive at the perinuclear region, and enter the nucleus (city center) through a nuclear pore (tunnels). During the journey, some passengers may be let off, and others may hop aboard, before the cable car reaches its destination in the nucleus.

Cell volume for asynchronously dividing endothelial cells is 882 ± 234 to 1835 ± 282 μm^3^ [36]. Meanwhile, cells with characteristic volumes of 2000–4000 μm^3^ typically contain about 10^10^ protein molecules per cell (2–4 × 10^6^ protein/μm^3^) [37]. Thus, the intracellular space is crowded with numerous molecules with varying types of membrane organelles (Figure 1b envisions the busy intracellular space where TMEDs are in motion, bypassing various organelles). TMED trafficking from the cell surface permits a well-organized transmission of extracellular stimuli to the intracellular signaling, including gene expression regulation.

## 3. Research Directions

TM4SF1-mediated biochemical and cellular activities observed in endothelial cells are also noted in other cell types that naturally express high TM4SF1 levels or are transformed to overexpress TM4SF1 [10,16]. This implies that, regardless of cell type, TM4SF1 conducts a common signaling program to regulate migration and intercellular interactions once TM4SF1 expression has exceeded the threshold needed to form TMEDs. This threshold may be surpassed by a variety of human diseases, such as cancers. Thus, understanding the mechanisms behind TM4SF1 expression regulation and TMED-mediated cell signaling may aid in the development of therapies for diseases. Potential research directions are summarized in Table 1 and the text below:

1. TM4SF1 transcriptional regulation. Why is TM4SF1 expression restricted to endothelial and mesenchymal stem cells among normal cell types, but ubiquitous among the tumor cells of solid cancers? We have identified ERG as the primary transcription factor regulating TM4SF1 expression in cultured endothelial cells (responsible for 60–70% of TM4SF1 expression; paper in preparation). The transcriptional regulation of TM4SF1 in stem cells of mesenchymal or cancer origin, or during the epithelial-to-mesenchymal transition into invasive and metastatic tumor cells [5,38,39], remains largely unknown, as is the mechanism of TM4SF1 suppression in most cell types.
2. TMED formation. The density of TMED on the cell surface is directly affected by the expression level of TM4SF1 [8,13]. We have noted that the cultured endothelial cells contain 0.5–1 × 10^6^ copies of TM4SF1 protein molecules on their cell surface [40]; however, the minimum number of TM4SF1 proteins needed on the endothelial cell surface to form TMED has not yet been quantified. Such knowledge will resolve a key difference in endothelial cell biology between the quiescent non-proliferative state versus the angiogenic proliferative state.
3. TMED recruited passenger molecules. Mapping the proteins recruited to TMEDs is highly desirable as it will (i) permit a better understanding of how cell signals are transmitted from cell surface to nucleus for gene expression regulation, (ii) elucidate proteins involved in intercellular interactions via nanopodia for the orchestration of blood vessel development, and (iii) open a new gateway for understanding the oncogenic transformation of epithelial cells to tumor cells [38,39].
4. TMED internalization and nuclear entry. TMED recruitment of HDAC6, a microtubule-associated deacetylase that shuttles to the nucleus by interacting with importins [41,42,43], likely plays a critical role in the process of TMED internalization along microtubules and ultimate arrival in the nucleoplasm. The observation that αTubulin is highly acetylated after TM4SF1 knockdown, despite normal HDAC6 expression levels, indicates that HDAC6 deacetylation activity is dependent on its transportation via TMEDs in endothelial cells [1]. HDAC6 is upregulated in various cancer types [44] and promotes cancer cell metastasis [45,46], suggesting a potential connection between HDAC6 and TM4SF1 in tumor cells.
5. Nanopodia-mediated intercellular interactions and communications. Nanopodia play a vital role in intercellular interactions and molecule/organelle trafficking, in both homotypic endothelial–endothelial and heterotypic endothelial–mesenchymal and endothelial–tumor cell interactions [10,11]. Mitochondria are transferred via tunneling nanotubes in mesenchymal stem cells [47]; we anticipate that these nanotubes form from nanopodia
6. The role of TM4SF1 in diseases of pathological angiogenesis. Many human diseases are initiated by a dysfunctional endothelium [48,49]. Conditional TM4SF1 knockout in endothelial cells in mice in vivo is the next step toward understanding not only the role of TM4SF1 in blood vessel development but also how it potentially orchestrates disease progression in vivo.
7. Tetraspanin-enriched microdomains (TEMs) versus TMEDs. Some genuine tetraspanins are ubiquitously expressed, while others are exclusively expressed [50]; some are reported to be endocytosed via clathrin-mediated endocytosis with an ultimate destination of endosomes–lysosomes or exosomes [20,21,51], while others internalize into the nucleus [52] with some forming migrasomes for intercellular communications [53,54]. How cells utilize expression level differences in these tetraspanins, both genuine and atypical, to achieve their cellular functions needs to be further explored.

### Therapeutic Implications of TMED Trafficking

TM4SF1’s high expression in proliferating endothelial cells and tumor cells of solid tumor origin, and low or no expression in most other cell types, positions TM4SF1 as an attractive target for solid tumor treatment. An L6-radioimmunoconjugate was tested in phase 1 radioimmunotherapy trials from 1990 to 1997 and generated a 60% overall response rate with negligible toxicity to normal vasculature [55,56,57,58]. TM4SF1’s ability to internalize from the cell surface to the nuclear compartment suggests antibody drug conjugates (ADCs) as the appropriate drug modality [40]. Currently, a single TM4SF1-directed ADC, sponsored by Angiex, is in phase 1 clinical trials for the treatment of solid tumors.

## 4. Conclusions

The cable car concept of molecular trafficking explains how, given the crowded nature of intracellular compartments, molecules can be distributed robustly and precisely to their site of action. Better understanding of TM4SF1’s cargo distribution process, as stated in “Research Directions”, will enhance our knowledge of blood vessel formation in normal and pathological contexts, and also improve our understanding of the oncogenic epithelial–mesenchymal transition. If cable car molecular trafficking is universally employed to enable targeted cell signaling, then other tetraspanins are likely to serve a similar transport function in cells that do not, or weakly, express TM4SF1. In short, “cable car”-like targeted molecular trafficking may be essential for normal biological function and may play important roles in pathology while providing opportunities for precision drug design, but research is needed to elucidate those functions, roles, and opportunities.

## Figures and Tables

**Table 1:** TMED Research Directions.

	Research Directions:	Investigates the Mechanisms Regarding:
**1**	TM4SF1 transcriptional regulation	The regulation of TM4SF1 transcription in angiogenic endothelial cells, mesenchymal stem cells, and tumor cells.
**2**	TMED formation	The number of TM4SF1 protein copies needed to enable the recruitment of proteins to form a TMED.
**3**	TMED recruited passenger molecules	The identity of proteins recruited to TMED, and the TM4SF1-enabled signaling networks involved in blood vessel formation and the oncogenic transformation of epithelial cells.
**4**	TMED internalization and nuclear entry	The destinations of TMED passenger molecules and their influence on cell biology.
**5**	Nanopodia-mediated intercellular interactions and communications	Nanopodia formation, and roles of nanopodia in intercellular interactions and molecule and organelle trafficking.
**6**	The role of TM4SF1 in diseases of pathological angiogenesis	TM4SF1 expression variations in blood vessel development in mice in vivo through the conditional knockout approach.
**7**	Tetraspanin-enriched microdomains (TEMs) versus TMEDs	The coordination between TEMs and TMEDs for the maintenance of cellular functions.

## Data Availability

No new data were created or analyzed in this study. Data sharing is not applicable.

## Abbreviations

TM4SF1, Transmembrane-4 L Six Family member 1; TMED, TM4SF1 enriched microdomain; ADC, antibody drug conjugate; MTOC, microtubule organizing center; PLCγ1, phospholipase C, gamma 1; HDAC6, histone deacetylase 6; VEGF-A, vascular endothelial growth factor-A; VEGFR2, VEGF receptor 2.
